# The clinical characteristics and prognosis of ABPA are closely related to the mucus plugs in central bronchiectasis

**DOI:** 10.1111/crj.13111

**Published:** 2019-12-05

**Authors:** Hai‐Wen Lu, Bei Mao, Ping Wei, Sen Jiang, Hong Wang, Cheng‐Wei Li, Xiao‐Bing Ji, Shu‐Yi Gu, Jia‐Wei Yang, Shuo Liang, Ke‐Bin Cheng, Jiu‐Wu Bai, Wei‐Jun Cao, Xin‐Ming Jia, Jin‐Fu Xu

**Affiliations:** ^1^ Department of Respiratory and Critical Care Medicine Shanghai Pulmonary Hospital, Tongji University School of Medicine Shanghai China; ^2^ Department of Radiology Shanghai Pulmonary Hospital, Tongji University School of Medicine Shanghai China

**Keywords:** allergic bronchopulmonary, aspergillosis, prognosis, radiology

## Abstract

**Introduction:**

The characteristics of Allergic Bronchopulmonary Aspergillosis (ABPA) based on its radiological classification is still unclear.

**Objectives:**

To investigate the clinical significances of ABPA patients with central bronchiectasis (ABPA‐CB) by different radiological classifications of mucus plugs.

**Methods:**

ABPA‐CB patients from a pulmonary hospital between 2008 and 2015 were retrospectively included and analysed. According to the chest imaging in their first visit to physician, the ABPA‐CB patients were divided into two groups based on the presence of high‐attenuation mucus (HAM) or low‐attenuation mucus (LAM). The primary endpoint was ABPA relapse within 1 year since the glucocorticoid withdrawal. The relationship between the imaging findings and the clinical prognosis was illuminated.

**Results:**

A total of 125 ABPA patients were analysed in this study. Compared to the LAM group, the HAM group presented higher blood eosinophil cells counts, higher rates of *Aspergillus* detection isolated in sputum and expectoration of brownish‐black mucus plugs, more affected lobes and segments, poorer pulmonary function and higher rate of relapse.

**Conclusions:**

The clinical characteristics and prognosis of ABPA‐CB patients are closely related to its radiological phenotype of mucus plugs in the central bronchiectasis. Clinicians should promote a diversity of personalized treatments for different patients with different radiological characteristics.

## 1 INTRODUCTION

Allergic bronchopulmonary aspergillosis (ABPA) is a respiratory disease characterized by a hypersensitive immune response to *Aspergillus* infection, whose pathogenesis dependents on individual gene susceptibility.^1^ Recently, we identified the homozygous CARD9 mutation encoding S12N in patients with ABPA and revealed the activation of RelB and production of IL‐5 in peripheral blood mononuclear cells from these patients.^2^ ABPA most commonly occurs as a comorbidity in patients with asthma and cystic fibrosis, and it is estimated to complicate the disease course in 1%‐12.9% of patients suffering from chronic persistent asthma^3^, ^4^, ^5^ and in 38.6% of patients admitted to the intensive care unit.^6^ A study of 200 Chinese bronchial asthma patients showed that 11 (11/200, 5.5%) presented a positive *Aspergillus* skin prick test, including five (5/200, 2.5%) cases diagnosed with ABPA.^7^ Taking into account that ABPA exists in asthma and/or bronchiectasis patients, implies that ABPA is not a rare disease in China and that its morbidity has been underestimated for a long time.

Radiologically, ABPA is characterized by CB with recurrent episodes of mucus plugging.^8^, ^9^ Some cases exhibit HAM.^2^, ^10^, ^11^, ^12^ Studies have shown that ABPA patients with different radiological findings can exhibit significant clinical heterogeneity.^1^ Therefore, critical classification and analysis of ABPA patients based on their radiological results are essential to improve treatment and prognosis in the ABPA‐CB patients.

In the present study, we analysed 125 patients diagnosed with ABPA‐CB within the last 7 years; 101 (80.8%) participated in the prospective follow‐up. The patients were divided into two groups based on the presence of HAM. Immune indices, pulmonary function tests, treatment and prognosis were statistically analysed. We also investigated the clinical correlations and distinctions between ABPA‐CB patients with different types of radiological findings to increase our knowledge of the disease, better guide treatment and predict outcomes.

## 2 MATERIALS AND METHODS

### 2.1 Subjects

We analysed the clinical data of ABPA‐CB patients at Shanghai Pulmonary Hospital, Tongji University, from January 2008 to December 2015. All patients were diagnosed according to the *Aspergillosis* treatment guidelines developed by the Infectious Diseases Society of America in 2008.^13^ The diagnosis of all patients was confirmed by three respiratory physicians and the CT scans were evaluated by one radiologist and one respiratory physician. The study protocol was approved by the Ethics Committee of Shanghai Pulmonary Hospital and written informed consent was obtained from all patients.

### 2.2 Study and follow‐up protocols

**Figure 1:**
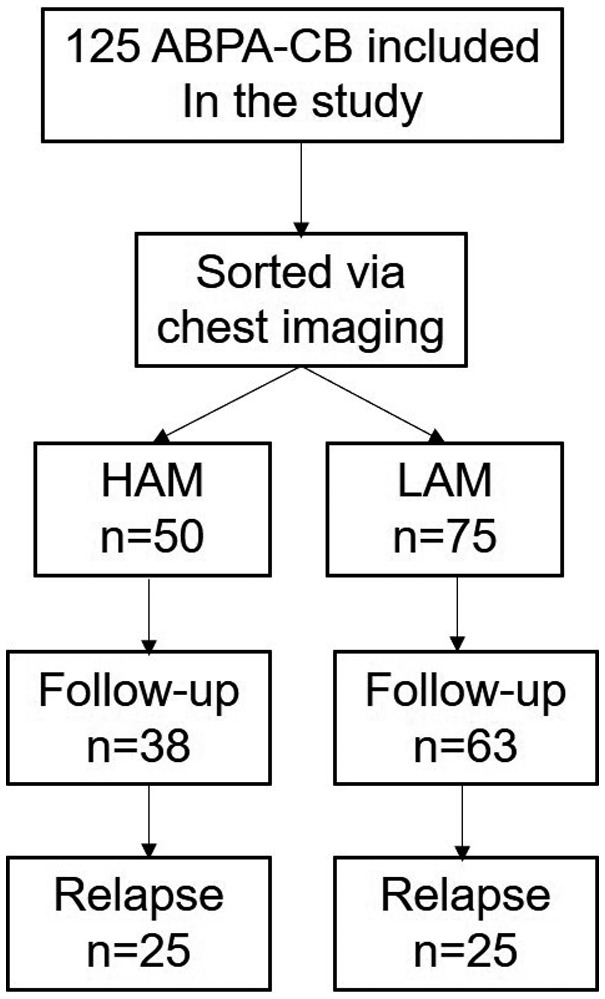
Flow chart. Study design and subject disposition

The 125 patients diagnosed with ABPA‐CB were confirmed with complete information in accordance with diagnosis standards and were included in this study. Data of demographics, cigarette smoking, history of anti‐tuberculous therapy, respiratory symptoms, laboratory tests (including serological findings), imaging findings, spirometry, treatment and outcome were collected from the hospital medical records. After they discharged from the hospital, patients visit the clinic every 4‐6 weeks to reassess the condition and adjust the drug dose according to the chest imaging and blood test, such as total IgE, blood cell count and the function of liver and kidney. The glucocorticoid was withdrew after comprehensive evaluation of the condition by physicians. After the drug withdrawal, patients was followed up every 3 months to assess the condition according to the chest imaging and total IgE. Patients were identified as having relapsed if total IgE reached twice baseline levels with a deterioration in the clinical and the radiological outcomes.^14^, ^15^, ^16^, ^17^ The primary endpoint was ABPA relapse within 1 year since the glucocorticoid withdrawal. A total of 101 patients participated in monthly follow‐up from December 2011, and relevant patient information was obtained at regular clinic visits (every 4‐6 weeks) and by telephone until the final follow‐up on December 31, 2015. The detailed research and follow‐up process is shown in Figure 1. Patients were divided into ABPA‐CB‐LAM and ABPA‐CB‐HAM based on high‐resolution chest computed tomography (HRCT) in their first visit to physicians. The criteria for CB were based on the radiographic evidence of bronchiectasis limited to the medial half of the lung.^18^, ^19^ Presence or absence of HAM (70‐90HU) with attenuation higher than the skeletal muscles was observed.^2^, ^12^ Clinical characteristics and prognosis were compared and analysed between the two groups.

### 2.3 Statistical analysis

The statistical package SPSS (version 16.0; SPSS, Chicago, IL, USA) was used for the statistical analyses. Graphs were drawn in GraphPad Prism (version 5; GraphPad Software, San Diego, CA, USA). The data were tabulated as mean ± standard deviation in the case of quantitative variables and as absolute numbers and percentages in the case of qualitative variables. In the bivariate analysis, the *t* test for independent variables was used to analyse variables that were normally distributed, such as eosinophil counts. The Mann‐Whitney U test was used to analyse variables that were not normally distributed, such as the measure of the pulmonary function. Qualitative variables were compared using the chi‐squared test, such as the number of expectorations of brownish‐black mucus plugs and the frequency of relapse. Significance was assumed at *P* < .05.

## 3 RESULTS

**Figure 2:**
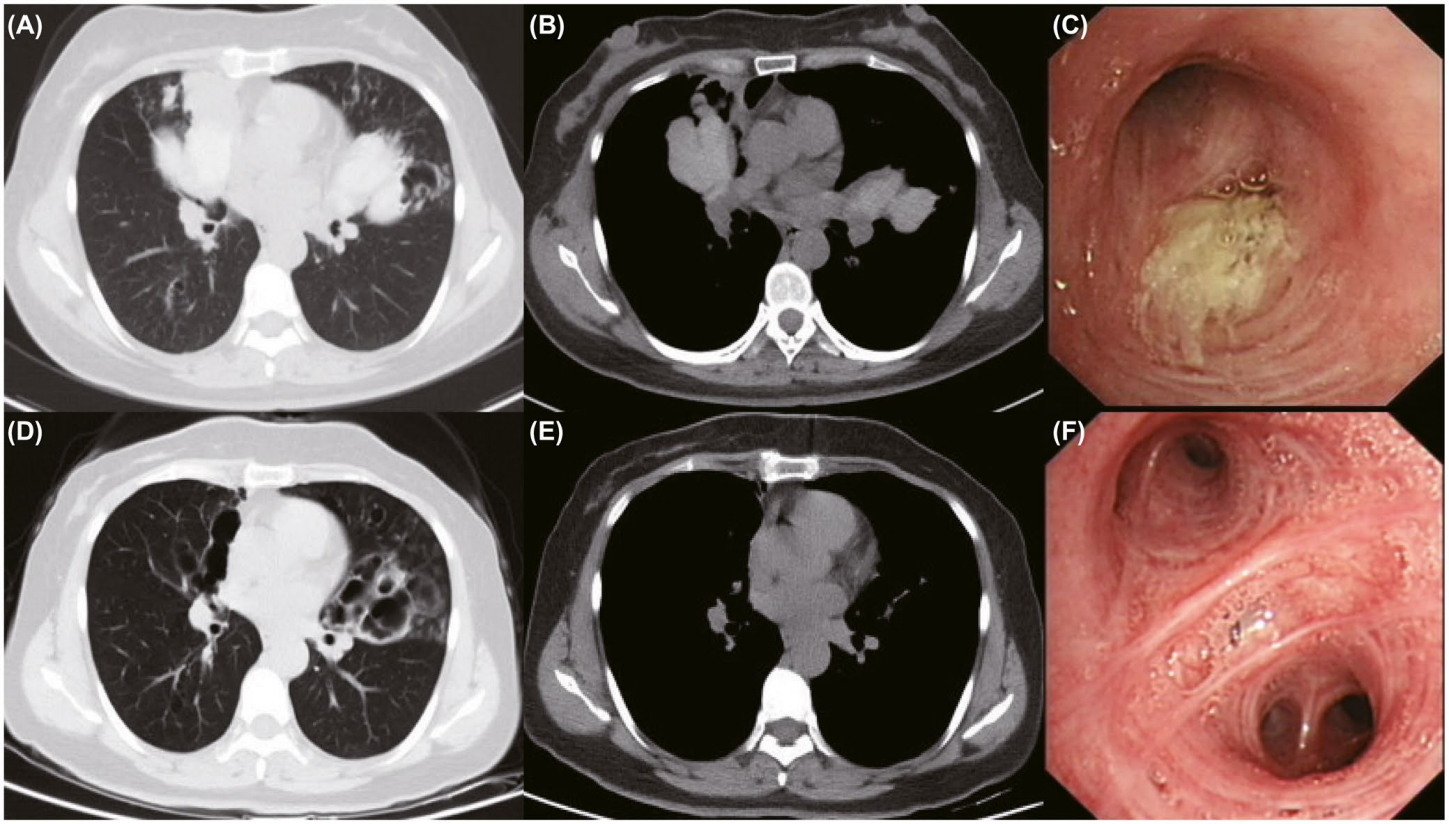
CT scans and bronchoscopy images of HAM before and after the comprehensive treatment. (A,B,C): before the treatment. (D,E,F): after the treatment

**Figure 3:**
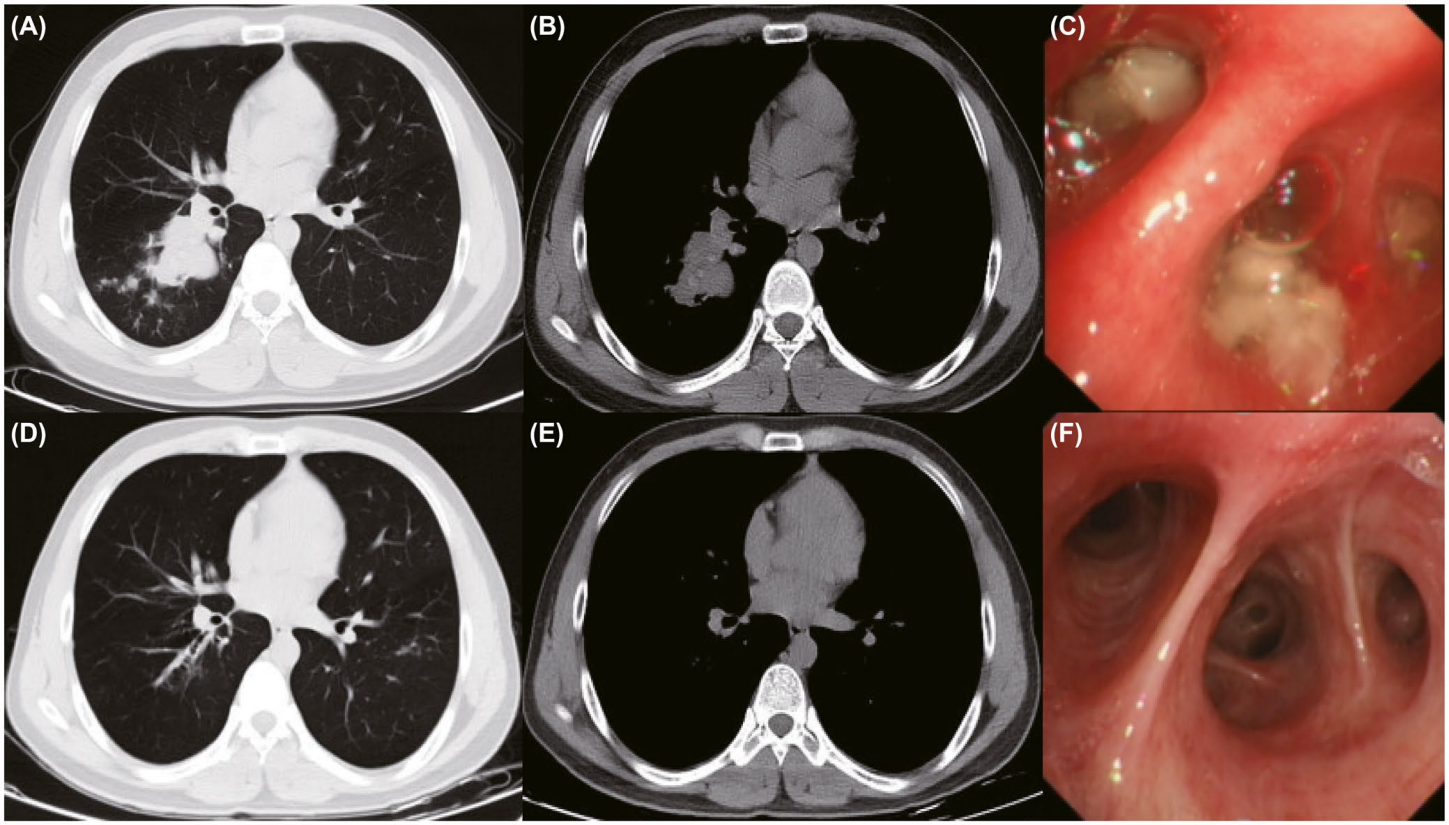
CT scans and bronchoscopy images of LAM before and after the comprehensive treatment. (A,B,C): before the treatment. (D,E,F): after the treatment

Among patients with significant mucoid impaction, mucus density measurements manifested as high attenuation CT values of 86 Hounsfield units (Hu) to 129 Hu. CT scans and bronchoscopy images of patient with HAM or LAM before and after the comprehensive treatment are shown in Figures 2 and 3. Of the 125 ABPA‐CB patients, 101 fully completed the follow‐up and 50 patients relapsed. Fifty patients were identified for the HAM subgroup of ABPA‐CB.

The demographic details, clinical features, serological findings and spirometry results in ABPA‐CB groups were shown in Table 1. HAM group presented younger age than the LAM group. The HAM group also presented higher *Aspergillus* detection rate in the sputum samples and more expectoration of brownish‐black mucus plugs. Eosinophil counts were higher in the HAM group than the LAM group. In addition, spirometry data showed that the HAM group had lower FEV1% and PEF% than the LAM group, which means greater damage to the pulmonary function. On CT images, the HAM group presented more extensive invasion of the pulmonary structure. In terms of prognosis, there was a higher relapse rate in the HAM group than the LAM group.

**Table 1:** Clinical, spirometric and serological differences and outcomes in patients with ABPA‐CB

	ABPA‐CB		*P*‐value
	HAM	LAM	
N	50	75	
Demographic details			
Age, years (x ± s)	40.2 ± 14.1	47.5 ± 12.5	.003
Gender, no. of males (%)	25 (50%)	35 (46.7%)	.72
History			
Hemoptysis, n (%)	5 (10%)	8 (10.7%)	.91
Expectoration of brownish‐black mucus plugs, n (%)	33 (66%)	31 (41.3%)	.007
Laboratory examination			
Eosinophil count, cells/mL (10^9^/L, x ± s)	2.181 ± 0.77	1.52 ± 0.69	<.001
*Aspergillus* detection in sputum specimens, n (%)	26 (52%)	20 (26.7%)	.004
Specific IgE against aspergillus > 0.35 KU/L, n (N^*^)	39 (39)	61 (63)	1
Skin test positive, n (N^*^)	29 (33)	38 (45)	0.919
Spirometry			
FEV1% (x ± s)	58.7 ± 14.8	65.9 ± 16.2	.03
FVC% (x ± s)	82.4 ± 10.9	82.4 ± 9.8	.99
FEV1/FVC% (x ± s)	63.3 ± 14.2	64.1 ± 14.4	.78
PEF% (x ± s)	57.1 ± 14.7	65.6 ± 13.4	.005
HRCT findings			
CT value	103.7 ± 15.1	40.0 ± 20.2	<.001
No. of lobes (x ± s)	3.0 ± 0.9	2.5 ± 0.9	.001
No. of segments (x ± s)	5.8 ± 1.8	4.9 ± 1.7	.006
Clinical outcome (n = 101)			
N	38	63	
No. of relapses (%)	25 (65.8%)	25 (39.7%)	.01

Abbreviations: ABPA, allergic bronchopulmonary aspergillosis; CB, central bronchiectasis; HAM, high‐attenuation mucus; IgE, immunoglobulin E; FEV1, forced Expiratory Volume in 1s; FVC, forced vital capacity; PEF, peak expiratory flow.

N^*^: The number of patients who were tested.

Of the 125 ABPA‐CB patients, 101 fully completed the follow‐up and 50 patients relapsed. The clinical, serological and spirometric data in ABPA‐CB patients according to the disease relapse were shown in Table 2. The relapsed patients showed higher eosinophil counts, expectoration of brownish‐black mucus plugs and mucus density values, as well as higher proportion of HAM patients compared to the patients without any relapse. Factor associated with the relapse of ABPA‐CB was eosinophil count (OR 1.001, *P* = .04) according to the regression analysis. Eosinophil count in blood was an independent risk factor of the disease relapse in ABPA patients with central bronchiectasis.

**Table 2:** Comparisons of patients with ABPA‐CB based on relapse or not

	Relapse	Non‐relapse	*P*‐value
N	50	51	
Demographic details			
Age, years (x ± s)	44.0 ± 13.3	45.9 ± 14.3	.49
Gender, no. of males (%)	28 (56%)	25 (49%)	.48
History			
Hemoptysis, n (%)	5 (10%)	6 (11.8%)	.78
Expectoration of brownish‐black Mucus plugs, n (%)	33 (66%)	21 (41.2%)	.01
Anti‐tuberculosis	27 (54%)	21 (41.2%)	.20
Laboratory examination			
Eosinophil count, cells/mL (10^9^/L, x ± s)	1.98 ± 0.76	1.52 ± 0.74	.003
*Aspergillus* detection in sputum Specimens, n (%)	22(44%)	14(27.5%)	.08
Spirometry			
FEV1% (x ± s)	60.1 ± 14.1	67.1 ± 17.5	.06
FVC% (x ± s)	82.6 ± 11.1	83.1 ± 11.1	.83
FEV1/FVC% (x ± s)	62.0 ± 11.8	67.4 ± 15.0	.09
PEF% (x ± s)	59.8 ± 14.4	65.4 ± 14.9	.10
HRCT findings			
CT value	75.1 ± 33.0	54.8 ± 32.9	.003
No. of lobes (x ± s)	2.78 ± 0.95	2.49 ± 0.95	.13
No. of segments (x ± s)	5.3 ± 1.72	5.0 ± 1.79	.42
ABPA‐HAM	25 (50%)	13 (25.5%)	.01

Abbreviations: ABPA, allergic bronchopulmonary aspergillosis; HAM, high‐attenuation mucus; IgE, immunoglobulin E; FEV1, forced Expiratory Volume in 1s; FVC, forced vital capacity; PEF, peak expiratory flow; HRCT, high resolution chest computed tomography.

**Figure 4:**
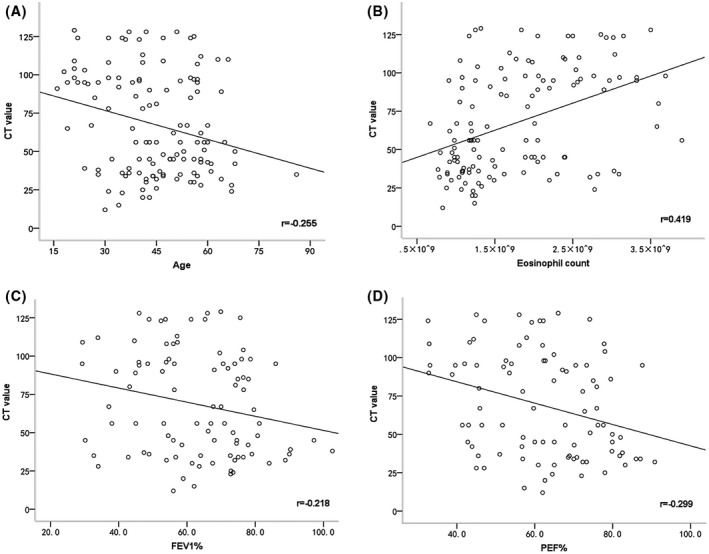
The scatter diagrams associated with the CT value of mucus in ABPA‐CB patients. The value of r represents the correlation coefficients between the CT value of mucus and age (A), eosinophil count on blood (B), FEV_1_% (C) and PEF% (D). The lines represents the relation trend of these indicators

The correlation coefficients between the CT value of mucus and clinical characteristics were shown in Figure 4. Factor positively correlated with the CT value of mucus was eosinophil counts in blood (*r* = .419, *P* < .001). Factors negatively correlated with the CT value of mucus were age (*r* = −0.255, *P* = .004), FEV_1_% (*r* = −0.218, *P* = .037) and PEF% (*r* = −.299, *P* = .004).

## 4 DISCUSSION

ABPA is a complex disorder caused by a hypersensitive immune reaction to antigens that are released by *Aspergillus* species, most often *A. fumigatus.* 20, 21 The common symptoms include wheezing, haemoptysis, low‐grade fever, productive cough^1^ and notably the expectoration of brownish‐black mucus plugs, which is considered to be a characteristic clinical manifestation of the disease.^3^, ^8^ Among the 125 patients in our study, 64 (51.2%) had a history of brownish‐black mucus plugs, which is comparable to previous studies.^22^ Because of the insidious onset of ABPA, its clinical symptoms are atypical and can often be mistaken for tuberculosis, bronchiectasis and pulmonary arteriovenous fistula.^23^, ^24^ Unrecognized or poorly treated ABPA leads to airway damage, bronchiectasis, and/or pulmonary fibrosis, eventually resulting in significant morbidity and mortality.^2^ In order to improve diagnostic accuracy, more clinical examinations should be carried out for patients with symptoms typical of ABPA, such as brownish‐black mucus plugs and refractory wheezing.

One common indicator for the diagnosis of ABPA is the patient’s peripheral blood eosinophil count exceeding 1000 cells/μL.^1^ However, as many as 60% of ABPA patients present eosinophil counts <1000 cells/μL at diagnosis.^25^ In our study, 12 patients (9.6%) had eosinophil counts <500 cells/μL, further indicating that the use of eosinophil count exceeding 1000 cells/μL as a screening criterion for ABPA may be conducive to misdiagnosis.^26^ In this research, the eosinophil count was significantly higher in ABPA‐HAM group compared with ABPA‐LAM group.

High‐resolution chest CT is the preferred method of radiological investigation in ABPA patients, as it can detect bronchiectasis, mucoid impaction, centrilobular nodules, tree‐in‐bud opacities, mosaic attenuation and pleuropulmonary fibrosis.^27^, ^28^ Mucus impaction in ABPA is usually hypodense, but in some patients, it is known to appear denser than the paraspinal skeletal muscle, which this is defined as HAM. In our study, 125 cases presented CB with mucus plug incarceration, 50 (40.00%, 50/125) of which were found to have HAM, which is roughly in agreement with the proportions reported in the literature.^15^ We conclude that bronchiectasis affecting three or more lobes, centrilobular nodules and mucoid impaction are strongly suggestive of ABPA.

Analysis of radiographic results is essential to determine what further diagnostic steps should be taken and a clinician with no knowledge of the radiographic characteristics of ABPA can easily misdiagnose patients on the first visit. With recent advances in our understanding of ABPA, a growing number of scholars have realized the potential of ABPA radiographic classification to improve our knowledge of the disease and guide early diagnosis, treatment and prognosis.

In ABPA‐CB patients, the changes in the pulmonary structure at the onset of bronchiectasis allows *Aspergillus* to more easily colonize the lungs. The ensuing increase in antigen release causes the destruction of airway epithelial cells and activation of T‐lymphocytes, increasing total serum IgE levels and local eosinophilia, leading to an inflammatory response in the airway wall and surrounding tissue that eventually induces bronchospasm. Asthma patients should be routinely screened for ABPA (e.g., *Aspergillus*‐specific IgE levels or skin testing) to detect the disease before bronchiectasis occurs.

Patients with ABPA‐CB‐HAM presented a higher relapse rate than patients with ABPA‐CB‐LAM in this study. Factor associated with poor prognosis in ABPA‐CB patients was eosinophil counts. The results indicate that the degree of eosinophilic inflammation may closely correlate with the risk of relapse. This makes anti‐inflammatory treatment more important. Furthermore, patients with HAM had a high relapse rate (65.8%), which requires patients to regularly follow‐up. Goyal et al first described the occurrence of HAM in ABPA in 1992,^10^ which has been shown to affect approximately 20% of ABPA patients.^29^ The results of our study indicate that the presence of HAM is the most consistent marker of serological severity in the disease. The HAM group had both significantly higher peripheral eosinophil counts and more serious small airway ventilatory dysfunction (FEV1%, *P *= .03 and PEF%, *P *= .005) than the LAM group. The presence of HAM is likely to define a subgroup of patients with more severe inflammation and structural damage of the lungs. However, the presence of HAM was not significantly associated with the relapse of patients with ABPA‐CB according to the regression analysis. Multicentre studies and large sample sizes are needed to further confirm the relationship between HAM and disease prognosis.

A multivariate analysis by Agarwal et al found both CB and HAM to be independent predictors of frequent ABPA relapse (OR 3.41, 95%CI 1.45‐8.01 and OR 3.61, 95%CI 1.23‐10.61, respectively).^15^ In agreement with their results, we found that the relapse rates were higher in the ABPA‐CB‐HAM group than the LAM group, suggesting that HAM not only represents immunologically severe disease, but also identifies patients at risk of recurrent relapse. However, these patients also had more severe dysimmunity and more serious airway inflammatory responses, which may have led to more difficulty in controlling their symptoms.

The pathogenesis of HAM is not entirely clear, but the higher attenuation implies a more viscous type of mucus. HAM is speculated to represent the presence of calcium and/or other metallic ions within the thickened or desiccated mucus, resulting in airway damage, bronchiectasis, and/or pulmonary fibrosis, eventually leading to significant morbidity and mortality. Further prospective studies should identify which patients are particularly at‐risk and may require more intensive treatment protocols and closer monitoring.

In summary, the clinical characteristics and prognosis of ABPA‐CB patients are closely related to its radiological phenotype of mucus plugs in the central bronchiectasis of these patients. Clinicians should promote a diversity of personalized treatments for different patients with different radiological characteristics. More studies should be carried out to figure out the mechanisms of HAM formation in ABPA.

## Conflict of interest

All the authors do not have any possible conflicts of interest.

## Author Contributions

XJF and LHW contributed to the study design, data analysis and interpretation. WP, JS, WH, LCW, JXB, GSY, YJW, LS, CKB, BJW, CWJ contributed to the data collection. WP, JXM and MB contributed to the data analysis, interpretation and the writing of the paper.

## Ethics

The study protocol was approved by the Ethics Committee of Shanghai Pulmonary Hospital, and written informed consent was obtained from all patients. The number of ethics is K16‐275. A scanned copy of this file has been uploaded to the attachment.
